# Impact of pretransplant vitamin D deficiency on immune recovery and clinical outcomes in multiple myeloma patients undergoing autologous stem cell transplantation

**DOI:** 10.3389/fimmu.2025.1588919

**Published:** 2025-10-31

**Authors:** Amany R. Keruakous, Nabil Ghani, Shreya Desai, Christopher Terrell, Shahrzad Zamani, Linda Youn, Sindu Iska, Mai Keruakous, Zhu Cui

**Affiliations:** ^1^ Department of Hematology and Oncology, Augusta University, Augusta, GA, United States; ^2^ Georgia Cancer Center, Augusta University, Augusta, GA, United States; ^3^ Department of Internal Medicine, Philadelphia College of Osteopathic Medicine (PCOM), Philadelphia, PA, United States; ^4^ Department of Internal Medicine, St. Bernard Medical Center, Jonesboro, AR, United States; ^5^ Department of Acute Care Oncology Nursing, Rutgers Robert Wood Johnson University Hospital, New Brunswick, NJ, United States; ^6^ Faculty of Medicine, Ain Shams University, Cairo, Egypt

**Keywords:** multiple myeloma, immune reconstitution, vitamin D, post transplant outcomes, supportive care

## Abstract

This study examines the impact of pretransplant vitamin D deficiency on immune recovery and clinical outcomes in multiple myeloma patients undergoing autologous stem cell transplantation (ASCT). Given vitamin D’s known immunomodulatory effects, the research describes its influence on neutrophil and platelet engraftment, lymphocyte recovery, and overall response rates post-ASCT. By analyzing a retrospective cohort, the study highlights potential associations between vitamin D status and post-transplant immune reconstitution, contributing to understanding vitamin D’s role in optimizing treatment strategies for multiple myeloma patients. Multiple myeloma (MM), considered the second most common hematological malignancy, is a plasma cell neoplasm that causes morbidity and mortality through its effects on organs, organ systems, and immunity. Clinical manifestations of MM include renal dysfunction, hypercalcemia, osteolytic bone lesions, anemia (CRAB Criteria), pathological fractures, and immunosuppression. The majority of myeloma patients suffer from long-term immunoparesis, which means suppression of uninvolved immunoglobulins (Igs), and these findings have been reported to be associated with poor prognosis in patients with multiple myeloma (MM).

## Background

Multiple myeloma (MM), considered the second most common hematological malignancy, is a plasma cell neoplasm that causes morbidity and mortality through its effects on organs, organ systems, and immunity. Clinical manifestations of MM include renal dysfunction, hypercalcemia, osteolytic bone lesions, anemia (CRAB Criteria), pathological fractures, and immunosuppression. The majority of myeloma patients suffer from long-term immunoparesis, which means suppression of uninvolved immunoglobulins (Igs), and these findings have been reported to be associated with poor prognosis in patients with multiple myeloma (MM) (1).

Normal immunoglobulins (Igs) play an important role in adaptive immune response to infections. In MM patients, normal plasma cells were inhibited by the rapid proliferation of malignant plasma cells, which causes immunoparesis and makes patients vulnerable to infections (2).

Multiple myeloma (MM) management has changed significantly over the past 10 years. The use of three or four-drug combination induction therapy followed by autologous stem cell transplantation (ASCT) has become the standard of care for transplant-eligible patients with MM since randomized trials showed improved progression-free survival (PFS) and overall survival (OS) (3, 4).

The current focus in MM research is to understand the tumor microenvironment, mechanisms of immune escape, and the role of immune reconstitution after ASCT. MM progression is associated with loss of tumor-specific immunity, suggesting that immune surveillance plays a role in preventing disease progression (5, 6).

A previous prospective analysis studied immune reconstitution after ASCT. It showed that in multiple myeloma patients treated with ASCT, adequate immune reconstitution and high lymphocyte recovery after ASCT are independent predictors of MRD negativity (7).

Due to the effect of vitamin D on the immune system, we investigated its impact on the immune system recovery after transplant, seeking an opportunity to improve or expedite immune reconstitution after ASCT; given the association between immune reconstitution and overall disease outcomes, we sought this analysis to examine if there is an association between Vit D deficiency and immune recovery after ASCT.

Vitamin D is a fat-soluble vitamin that regulates calcium in metabolism and significantly influences the immune system. The significance of vitamin D in the context of multiple myeloma (MM) has become a focal point of attention, given its potential impact on clinical outcomes. Vitamin D affects osteoclast activity thereby maintaining both bone health and calcium homeostasis (8). Vitamin D receptors are found on immune cells like macrophages, CD4+ and CD8+ T cells. By activating these receptors, Vitamin D affects the immune system by reducing the pro-inflammatory cytokines and attenuating the pathological activation of Th17 cells (9).

Several studies have delved into the complex interplay between vitamin D levels and various aspects of MM, illuminating its role in this hematologic malignancy.

VitD deficiency is prevalent at approximately 40% among patients with MM and is associated with a higher number of plasma cells in the bone marrow at diagnosis (10, 11). Retrospective studies demonstrated a negative impact of VitD deficiency on skeletal burden and myeloma activity (12).

Besides vitamin D’s traditional impact on bone homeostasis, vitamin D also plays additional roles in cell regulation and immunoregulatory functions (13). Increasing evidence supports an immunomodulatory effect of vitamin D. It has been demonstrated that vitamin D receptor (VDR) is expressed in T- and B lymphocytes. Notably, VDR expression by these cells was only immunologically functional in active, proliferating cells, suggesting an anti-proliferative role for 1,25(OH)2 D in these cells (14).

This study examines the impact of pretransplant vitamin D deficiency on immune recovery and clinical outcomes in multiple myeloma patients undergoing autologous stem cell transplantation (ASCT). Given vitamin D’s known immunomodulatory effects, the research evaluates its influence on neutrophil and platelet engraftment, lymphocyte recovery, and overall response rates post-ASCT.

## Study design and method

A local institutional review board (IRB) approved a retrospective, single-center study. It included all patients aged 18 years or older who underwent high-dose Melphalan with autologous stem cell transplant (ASCT) for Multiple Myeloma between January 1, 2013, and December 1, 2020. The primary objective of the study was to evaluate the impact of vitamin D status on immune system reconstitution following autologous stem cell transplantation.

Patients were identified using our transplant database; extensive chart review was performed on all transplanted patients during the study. Patients were grouped based on their pre-transplant vitamin D levels. A serum level of 25-hydroxy vitamin D (25(OH)D) laboratory test was performed on all participants. Those with vitamin D levels below 30 ng/mL were categorized as “deficient,” while those with levels of 30 ng/mL or higher were considered “sufficient.” If multiple pre-transplant vitamin D measurements were available for a patient, the value closest to the transplant date was used for analysis.

Post-transplant immune reconstitution was assessed using several measures: time to neutrophil engraftment (defined as an absolute neutrophil count >500/mm³ for three consecutive days), time to platelet engraftment (defined as a platelet count >20,000/µL for 7 days without transfusion), and absolute lymphocyte count (ALC) on day 30 post-transplantation. Day 30 ALC was used as a surrogate marker for lymphocyte recovery and immune reconstitution, a measure employed in previous studies.

All patients were admitted by the day of cellular reinfusion (i.e., day 0 of transplant) and remained hospitalized until neutropenia and any adverse events were resolved. According to hospital policy, the Melphalan dose was calculated based on actual body weight, with rounding allowed within a 10% margin. Standard supportive care measures included a low microbial diet, growth factor support, and antiemetic prophylaxis, while antimicrobial prophylaxis was administered with antiviral, antifungal, and antibacterial agents.

The study reviewed various factors related to vitamin D status and its potential effects on immune reconstitution. It examined the relationship between vitamin D levels and several key clinical variables, such as disease status before and after ASCT, cytogenetic risk category, using the IMWG risk stratification criteria (by performing FISH and cytogenetics on marrow aspirate at the time of diagnosis), and disease response, using IMWG response criteria. All patients underwent disease restaging using bone marrow biopsy, myeloma serology, and PET CT day 90–100 after ASCT. The presence of CRAB criteria at the time of diagnosis was also considered. Additionally, the study assessed the differences in clinical outcomes, such as the time to neutrophil and platelet engraftment and the absolute lymphocyte count at days 30 and 90 post-transplant. This brief research is intended to describe the impact of vitamin D deficiency on the multiple variables listed above.

### Statistical methods

Descriptive statistics were used to summarize patient demographics and baseline clinical characteristics. Continuous variables were reported as means, medians, standard deviations (SD), minimums, and maximums. Categorical variables were summarized using frequencies and percentages.

Comparisons between groups (vitamin D deficient vs. non-deficient) were performed using: a) Independent samples t-test (for continuous variables such as time to neutrophil engraftment, platelet engraftment, absolute lymphocyte counts). b) Chi-square or Fisher’s exact tests (for categorical variables such as disease risk, anemia, hypercalcemia, bone disease, renal involvement, and overall response rates). c) Logistic regression was conducted to explore predictors of overall response rate (ORR) after autologous stem cell transplantation.

For all analyses, a two-sided P-value < 0.1 was considered statistically significant.

Statistical analyses were performed using SAS version 9.4.

## Results

### Baseline demographic

The study included 79 participants (**Table 1**). The mean age at diagnosis was 59.89 years, ranging from 38 to 75 years. The time to transplant from disease diagnosis had a mean of 418.29 days, ranging from 55 to 4308 days. The time to ANC recovery was 12.29 days, ranging from 10 to 19 days. The time to platelet recovery was 26.39 days, ranging from 10 to 511 days. The mean vitamin D level was 23.79 ng/mL, ranging from 4.2 to 90.3 ng/mL.

**Table 1:** Baseline demographics.

Variable	Value
Total Participants	79
Mean Age at Diagnosis	59.89 years (range: 38-75)
Time to Transplant	418.29 days (range: 55-4308)
Gender (Female/Male)	35 (44.3%)/44 (55.7%)
Disease Risk (High/Standard)	39 (54.17%)/33 (45.83%)
Disease Status Before Transplant	CR: 11 (13.92%), PR: 36 (45.57%), VGPR: 32 (40.51%)
Disease Status Post-Transplant	CR: 23 (29.11%), PR: 25 (31.65%), VGPR: 31 (39.24%)
Vitamin D Deficiency (Yes/No)	61 (77.22%)/18 (22.78%)

Among the participants, 35 (44.3%) were female, and 44 (55.7%) were male. Disease risk was categorized as high risk for 39 (54.17%) participants and standard risk for 33 (45.83%) participants, with 7 missing values. Prior to transplant, disease status was categorized as CR for 11 (13.92%), PR for 36 (45.57%), and VGPR for 32 (40.51%). Post-transplant disease status was categorized as CR for 23 (29.11%), PR for 25 (31.65%), and VGPR for 31 (39.24%).

Vitamin D evaluation was done within 100 days prior to ASCT. Vitamin D deficiency was present in 61 (77.22%) participants, while 18 (22.78%) did not have vitamin D deficiency. All participants who had vitamin D deficiency were prescribed replacement supplement.

### Immune reconstitution and Vitamin D deficiency

The mean time to ANC recovery was 11.83 days in participants without vitamin D deficiency and 12.43 days in those with deficiency (**Table 2**). The mean time to platelet recovery was 19.12 days in participants without vitamin D deficiency and 28.45 days in those with deficiency, showing a longer recovery time in the deficient group. The mean absolute lymphocyte count (ALC) on day 30 was higher in participants without vitamin D deficiency (1525 cells/µL) compared to those with deficiency (1193 cells/µL). On day 60, the mean ALC was 1613.9 cells/µL in participants without deficiency and 1530.9 cells/µL in those with deficiency.

**Table 2:** Vitamin D deficiency and immune reconstitution with expanded statistics.

Parameter	No Vitamin D Deficiency (n=18)	Vitamin D Deficiency (n=61)	P-Value
Time to ANC Recovery (days)	Mean 11.83, Median 12, SD 1.10, Min 10, Max 13	Mean 12.43, Median 12, SD 1.49, Min 10, Max 19	0.07
Time to Platelet Recovery (days)	Mean 19.12, Median 18, SD 5.35, Min 10, Max 29	Mean 28.45, Median 18, SD 63.66, Min 12, Max 511	0.26
Absolute Lymphocyte Count Day 30 (cells/μL)	Mean 1525, Median 1495, SD 984.5, Min 400, Max 3990	Mean 1193, Median 1090, SD 675.3, Min 90, Max 3000	0.10
Absolute Lymphocyte Count Day 60 (cells/μL)	Mean 1613.9, Median 1375, SD 980.2, Min 410, Max 3900	Mean 1530.9, Median 1440, SD 913.8, Min 250, Max 4450	0.74

**Figure 1:**
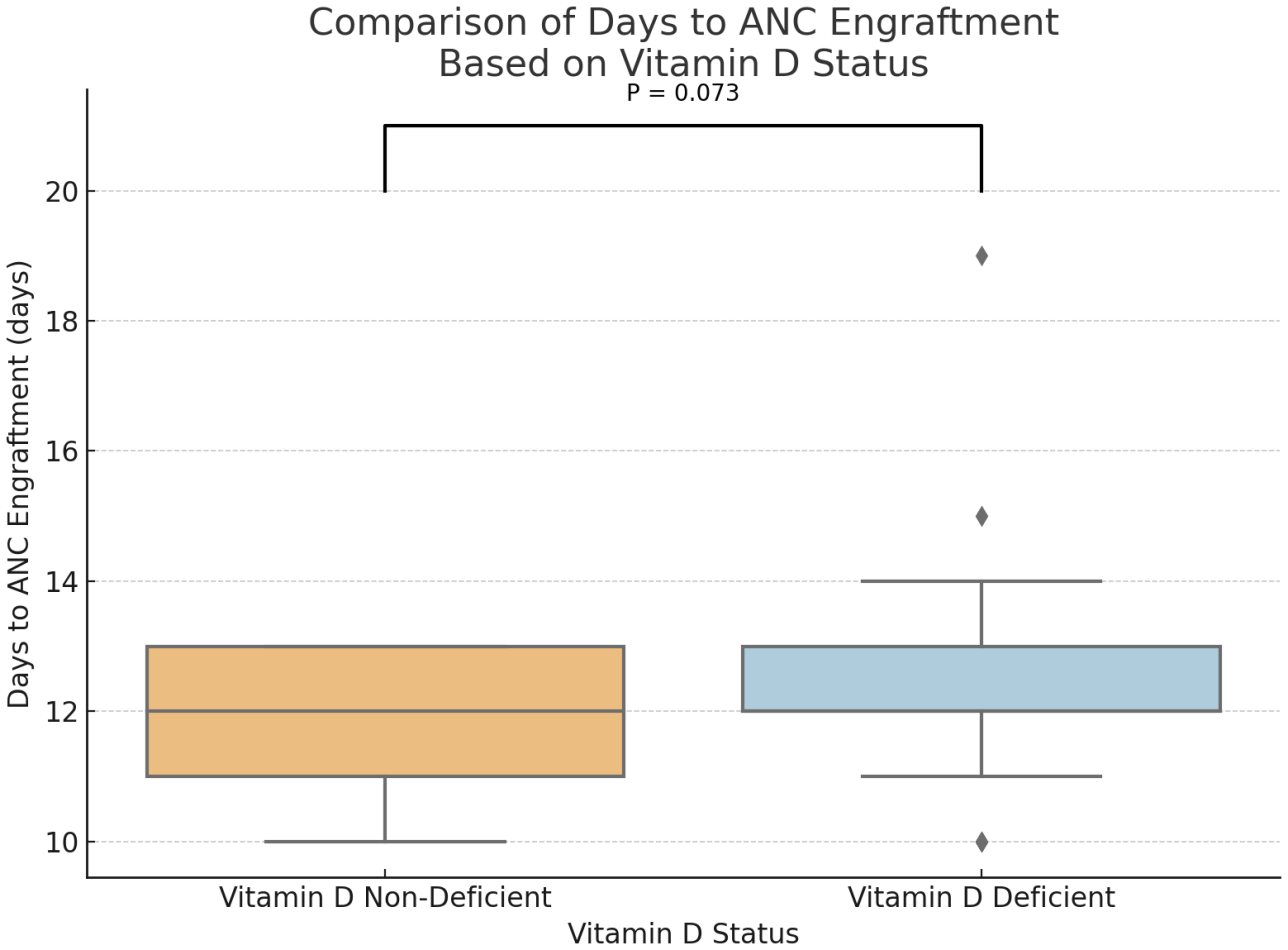
Comparison of Days to ANC Engraftment based on Vitamin D status. (p=0.073).

**Figure 2:**
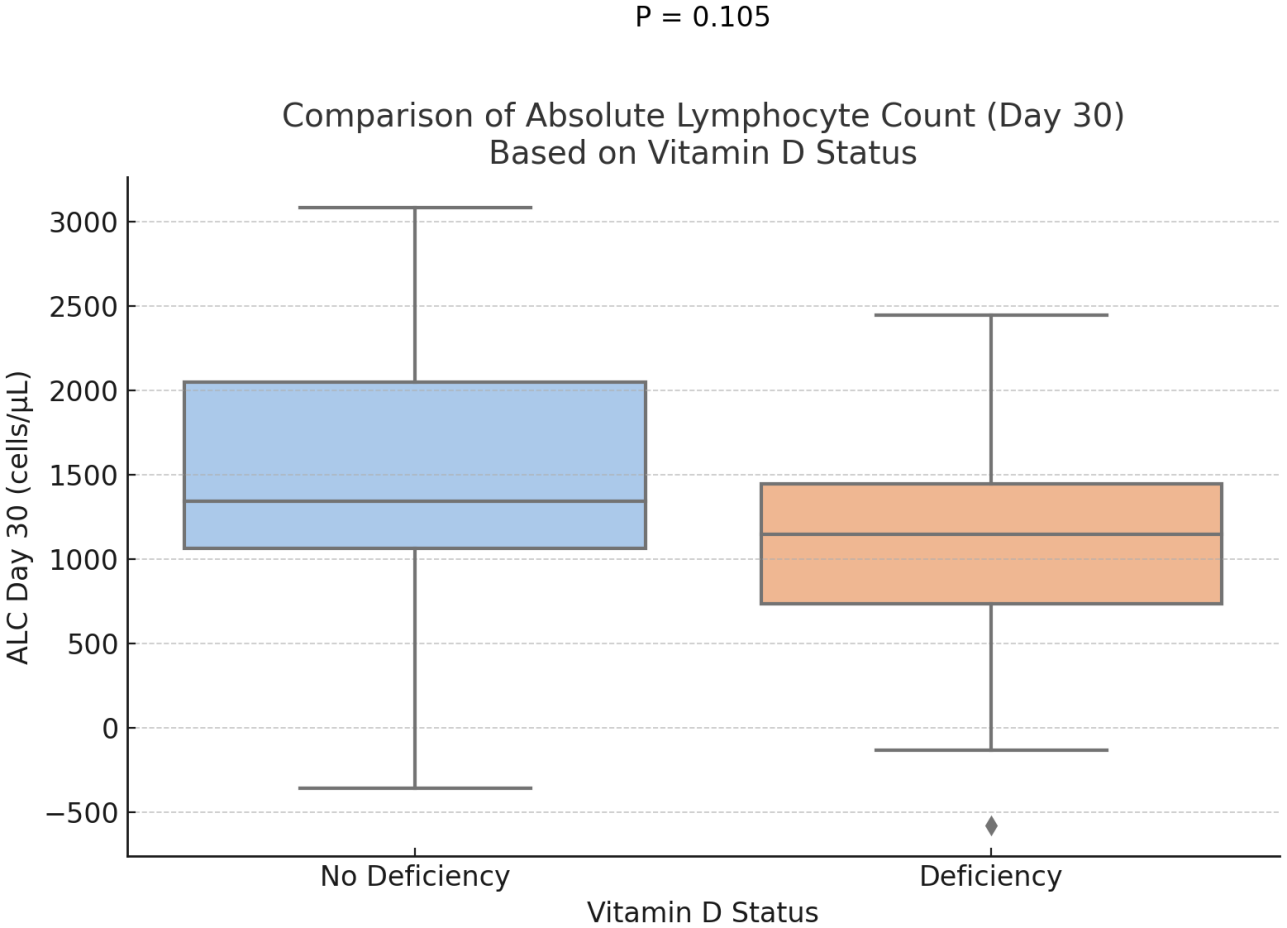
Comparison of day 30 absolute lymphocyte count based on Vitamin D status. (p=0.105).

Statistical analysis demonstrated a trend toward delayed neutrophil engraftment in vitamin D-deficient patients (P = 0.073) (**Figure 1**). And, a marginally significant difference in the absolute lymphocyte counts at days 30, with higher ALC in the group of participants without vitamin D deficiency (P = 0.1) (**Figure 2**). However, time to platelet engraftment was numerically longer in the vitamin D deficient group, this difference was not statistically significant.

### Vitamin D deficiency and disease burden at diagnosis

Among participants with vitamin D deficiency, 32 (82.05%) had a high disease risk, compared to 7 (17.95%) in the non-deficient group (**Table 3**). Among those without deficiency, 10 (30.30%) had standard disease risk, compared to 23 (69.70%) in the deficient group. Anemia was noticed to be more common in participants with vitamin D deficiency (20, 80.00%) compared to those without (5, 20.00%). Hypercalcemia at diagnosis was observed in 8 (80.00%) participants with vitamin D deficiency, compared to 2 (20.00%) without deficiency. Bone disease was present in 49 (79.03%) participants with vitamin D deficiency, compared to 13 (20.97%) without deficiency. Renal involvement, defined as serum creatinine > 2 or eGFR < 40 at time of diagnosis, was reported in 12 (70.59%) participants with vitamin D deficiency, compared to 5 (29.41%) without deficiency. Although, we noticed and increased incidence of high risk disease, anemia, hypercalcemia, as well as lytic bone lesions in the vitamin D deficient group, these observations were not included in our statistical analysis plan due to high incidence of missing data.

**Table 3:** Vitamin D deficiency and disease factors.

Factor	No Vitamin D Deficiency N=18	Vitamin D Deficiency N=61
High-Risk	7 (17.95%)	32 (82.05%)
Standard-Risk	10 (30.30%)	23 (69.70%)
Anemia	5 (20.00%)	20 (80.00%)
Hypercalcemia at Diagnosis	2 (20.00%)	8 (80.00%)
Bone Disease	13 (20.97%)	49 (79.03%)
Renal Involvement	5 (29.41%)	12 (70.59%)
Overall Response Rate (ORR)	55.6%	40.9%

### Vitamin D deficiency and overall response rate

Of particular interest was the observation of the ORR post-transplant, defined as the achievement of a very good partial response (VGPR) or better within 100 days following ASCT. The data indicated a numerically lower ORR among patients with vitamin D deficiency, with a rate of 40.9%, in contrast to the 55.6% observed among those with sufficient vitamin D levels.

## Discussion

This study highlights the widespread prevalence of vitamin D deficiency in multiple myeloma (MM) patients before autologous stem cell transplantation (ASCT), reinforcing prior findings by Gujarathi et al. (15) The deficiency was observed across genders and different age groups, suggesting it is a common issue in this patient population. Given vitamin D’s role in immune function and bone metabolism, our findings contribute to the growing body of literature exploring its implications in MM prognosis and treatment outcomes (16, 17).

### Vitamin D and immune reconstitution post-ASCT

Immune reconstitution is a key aspect of recovery in MM patients after ASCT, represented by neutrophil and platelet engraftment. Our study showed a marginally better rate of neutrophil engraftment and a statistically significant early platelet engraftment in patients who were not vitamin D deficient. Predictably, lymphocyte recovery was also delayed, as measured on day 30, although this did not reach statistical significance. These findings are consistent with prior research demonstrating vitamin D’s role in immune modulation and hematopoietic recovery.

Vitamin D regulates innate and adaptive immunity, influencing the activity of monocytes, dendritic cells, and lymphocytes. Studies have shown that vitamin D deficiency is associated with increased pro-inflammatory cytokine production, which may negatively impact immune recovery post-transplant. Furthermore, vitamin D receptors (VDR) are expressed on T and B lymphocytes, and their activation has been demonstrated to modulate immune responses, potentially reducing inflammation and promoting hematopoietic regeneration.

### Vitamin D and disease burden at diagnosis

**Figure 3:**
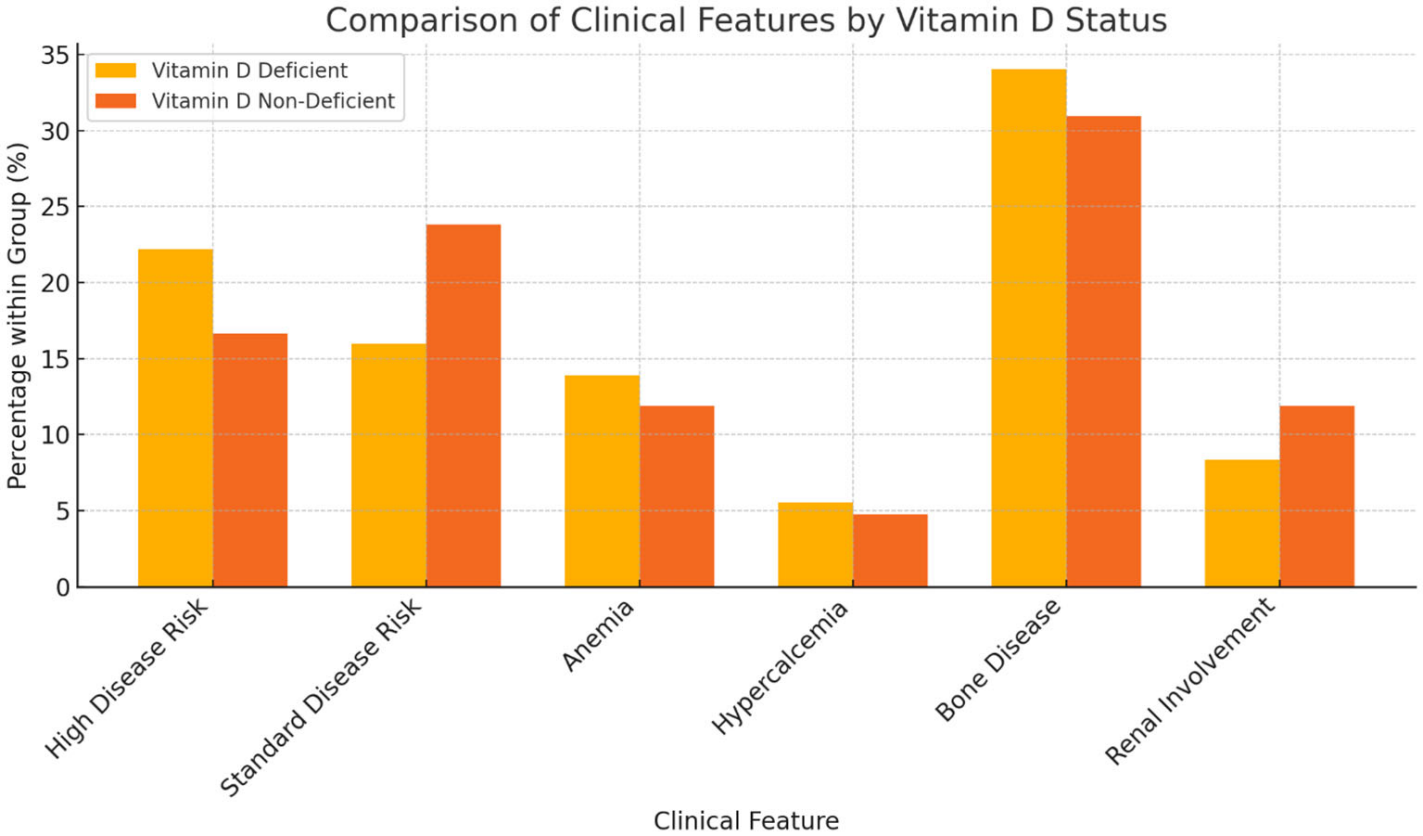
Comparison of Clinical features by Vitamin D status.

Our findings also highlight the association between vitamin D deficiency and increased disease burden at diagnosis. Patients with vitamin D deficiency were more likely to have high-risk disease features, including anemia, hypercalcemia, and bone disease. These observations align with previous retrospective studies that reported a negative impact of vitamin D deficiency on skeletal integrity and myeloma disease activity (18). The high prevalence of bone disease in the deficient group underscores vitamin D’s potential role in maintaining bone health and preventing MM-associated skeletal complications (**Figure 3**).

Several studies have indicated that vitamin D deficiency is prevalent in MM patients and may be linked to disease severity (17). The impact of vitamin D on bone homeostasis is well-documented, as it plays a crucial role in calcium metabolism and osteoclast regulation. Some studies suggest vitamin D deficiency may contribute to increased bone resorption, exacerbating skeletal complications in MM patients. Additionally, low vitamin D levels have been associated with greater tumor burden, more aggressive disease phenotypes, additionally more aggressive treatment-related toxicity (19), suggesting that vitamin D may play a role in MM pathogenesis beyond bone health.

### Vitamin D and overall response rate

The study also showed lower ORR in patients with vitamin D deficiency. Although this was not statistically significant, the trend was toward better ORR in vitamin D-sufficient patients. These results align with Ng et al.’s findings (17) and other scientists emphasizing the clinical significance of vitamin D status in MM (20). Given that immune reconstitution and tumor surveillance contribute to treatment efficacy, future studies should investigate whether correcting vitamin D deficiency before transplant can enhance response rates.

Existing literature has reported that vitamin D influences tumor microenvironment interactions, including suppressing myeloma cell proliferation and promoting apoptosis through its effects on various signaling pathways. Some studies have demonstrated that vitamin D supplementation may enhance the efficacy of anti-myeloma therapies, potentially improving treatment responses. However, additional research is required to determine the precise mechanisms by which vitamin D affects MM progression and treatment outcomes.

### Filling the gaps in knowledge

This study provides valuable insights into the correlation between vitamin D status and autologous transplant outcomes in MM patients. The findings resonate with several other research articles in the field, contributing to a nuanced understanding of the multifaceted role of vitamin D in MM management and prognosis. Unlike previous studies that focused primarily on vitamin D’s role in bone metabolism, our research emphasizes its impact on immune reconstitution and hematologic recovery. By demonstrating associations between vitamin D levels, platelet engraftment, and immune recovery, our findings suggest that routine vitamin D assessment and supplementation in transplant candidates may be a simple, cost-effective strategy to optimize post-transplant outcomes.

Our study reinforces the importance of checking vitamin D levels in every transplant patient during pre-transplant evaluation. This low-cost intervention could improve outcomes in this cohort of MM patients by addressing a potentially modifiable deficiency. Further research is needed to establish causality and determine whether vitamin D supplementation can enhance immune recovery and long-term survival in MM patients undergoing ASCT.

### Limitations

This study has several limitations. First, its single-center, retrospective design limits the generalizability of the findings. Second, the sample size is relatively small, which may have reduced the statistical power to detect significant associations. Due to the sample size and missing data across several variables, we were not able to perform a robust multivariate analysis to adjust for potential confounding factors, including race/ethnicity and other clinical covariates. While associations between vitamin D deficiency and features such as anemia, hypercalcemia, renal involvement, and bone disease were observed, these were descriptive in nature and not supported by formal multivariate statistical testing. Race and ethnicity data were not available for all participants and were therefore not included in the analysis, although most patients were of White descent. Third, confounding factors such as nutritional status, concurrent medications, and baseline immune function and post-transplant infection rates were not controlled, which may have influenced the results. Finally, the study did not evaluate the long-term effects of vitamin D deficiency correction on immune reconstitution and clinical outcomes. Future studies with larger, multi-center cohorts and prospective designs are needed to validate these findings and further investigate the impact of vitamin D supplementation in MM patients undergoing ASCT.

### Potential future considerations

Given the observed trends in our study, future research should focus on interventional studies that assess the impact of vitamin D supplementation on post-ASCT recovery. Prospective, randomized controlled trials evaluating pre-transplant vitamin D repletion strategies could help establish a causal relationship between vitamin D levels and improved immune reconstitution. Additionally, further investigations should explore the optimal threshold for vitamin D sufficiency in MM patients undergoing ASCT, as current clinical guidelines may not fully account for the immunomodulatory effects of vitamin D in this specific population.

Further prospective studies can be designed to divide vitamin D-deficient patients into two cohorts; outcomes after transplant can be compared between the cohort that receives and corrects vitamin D deficiency versus those that do not. More than one arm can be studied with different goals of vitamin D repletion that would help give specific recommendations in real-life clinical practice. Like Yellapragada et al., future studies can also stratify patients based on race and add more nuance to vitamin D deficiency and outcomes after repletion in MM patients post-transplant (21).

Future studies should also incorporate a more detailed analysis of immune cell subsets, cytokine profiles, and markers of immune activation to understand better the mechanisms by which vitamin D influences immune recovery. Longitudinal studies assessing long-term survival, progression-free survival, and quality of life in vitamin D-replete versus deficient patients would provide valuable insights into the broader implications of vitamin D status in MM treatment.

The current study’s findings regarding vitamin D levels among MM patients complement the existing research by further characterizing the vitamin D landscape in MM. In addition to these studies, several other research articles have contributed to understanding vitamin D’s role in MM and hematopoiesis, its effects on immune function, and its potential clinical applications (7, 14). Collectively, these studies highlight the complexity of vitamin D’s impact on MM and emphasize the importance of considering and addressing vitamin D deficiency in managing MM patients, particularly those undergoing ASCT, to optimize treatment strategies and enhance patient outcomes.

## Conclusion

In summary, this study underscores the potential influence of vitamin D status on post-transplant immune recovery and clinical outcomes in MM patients. While some associations did not reach statistical significance, the observed trends highlight the need for further research into the role of vitamin D in MM management. Routine screening and correction of vitamin D deficiency may represent a low-cost intervention to improve post-transplant recovery and overall patient outcomes.

## Data Availability

The original contributions presented in the study are included in the article/supplementary material. Further inquiries can be directed to the corresponding author.
